# Starburst amacrine cells form gap junctions in the early postnatal stage of the mouse retina

**DOI:** 10.3389/fncel.2023.1173579

**Published:** 2023-05-24

**Authors:** Takuma Maruyama, Toshiyuki Ishii, Makoto Kaneda

**Affiliations:** Department of Physiology, Graduate School of Medicine, Nippon Medical School, Tokyo, Japan

**Keywords:** starburst amacrine cells (SACs), gap junction, connexin (Cx), retinal ganglion cell (RGC), dopamine D1 receptor (D1R), visual experience, retina, development

## Abstract

Although gap junctional coupling in the developing retina is important for the maturation of neuronal networks, its role in the development of individual neurons remains unclear. Therefore, we herein investigated whether gap junctional coupling by starburst amacrine cells (SACs), a key neuron for the formation of direction selectivity, occurs during the developmental stage in the mouse retina. Neurobiotin-injected SACs coupled with many neighboring cells before eye-opening. The majority of tracer-coupled cells were retinal ganglion cells, and tracer coupling was not detected between SACs. The number of tracer-coupled cells significantly decreased after eye-opening and mostly disappeared by postnatal day 28 (P28). Membrane capacitance (Cm), an indicator of the formation of electrical coupling with gap junctions, was larger in SACs before than after eye-opening. The application of meclofenamic acid, a gap junction blocker, reduced the Cm of SACs. Gap junctional coupling by SACs was regulated by dopamine D1 receptors before eye-opening. In contrast, the reduction in gap junctional coupling after eye-opening was not affected by visual experience. At the mRNA level, 4 subtypes of connexins (23, 36, 43, and 45) were detected in SACs before eye-opening. Connexin 43 expression levels significantly decreased after eye-opening. These results indicate that gap junctional coupling by SACs occurs during the developmental period and suggest that the elimination of gap junctions proceeds with the innate system.

## 1. Introduction

Gap junctional coupling in the developing retina has many functional roles (Cook and Becker, 2009). The functional importance of gap junctions has been demonstrated in cell proliferation (Becker and Mobbs, 1999), cell migration (Pearson et al., 2005), neural circuit formation (Guldenagel et al., 2001; Deans et al., 2002), and the generation of synchronized activity, including retinal waves (Choi et al., 2021). In adulthood, gap junctions are used to improve the signal-to-noise ratio (DeVries et al., 2002), regulate the receptive field size (Shelley et al., 2006), control signal transmission (Deans et al., 2002; Veruki and Hartveit, 2002), and generate synchronized firing (Mastronarde, 1983; Brivanlou et al., 1998; Hu and Bloomfield, 2003; Trenholm et al., 2014). To exert a number of functions in the retina, the activity of gap junctions is controlled by dopamine (Hampson et al., 1992; Bloomfield and Volgyi, 2009; Roy and Field, 2019; Goel and Mangel, 2021) and nitric oxide (Daniels and Baldridge, 2011; Jacoby et al., 2018).

Starburst amacrine cells (SACs) release acetylcholine and GABA onto neighboring cells (O’Malley et al., 1992) and play an important role in the formation of direction selectivity in the adult retina (Yoshida et al., 2001; Amthor et al., 2002). To elucidate the mechanisms underlying direction selectivity, many researchers focused on neural computation mechanisms on the dendrites of SACs (Wei, 2018; Murphy-Baum et al., 2021), such as the distribution of channels (Ozaita et al., 2004; Gavrikov et al., 2006) and centrifugal signal transmission along dendrites (Euler et al., 2002; Lee and Zhou, 2006; Hausselt et al., 2007), as well as input-output relationships between SACs (Lee and Zhou, 2006), SACs and direction-selective ganglion cells (Lee et al., 2010; Briggman et al., 2011), SACs and bipolar cells (Kim et al., 2014; Ding et al., 2016), and SACs and other amacrine cells (Jain et al., 2022).

Since SACs are closely coupled with retinal functions from the developmental stage to adulthood, their morphological maturation has also been investigated. After the establishment of morphological findings (Masland and Tauchi, 1986) and their maturation (Wong and Collin, 1989; Kim et al., 2000; Zhang et al., 2005; Famiglietti and Sundquist, 2010), the molecular guide mechanisms of specific dendritic stratifications in SACs were subsequently examined as a morphological basis for direction-selective circuit formation in the developing stage (Sun et al., 2013; Whitney et al., 2014; Visser et al., 2015; Yonehara et al., 2016; Ray et al., 2018). However, in spite of a systematic analysis of the morphological properties of SACs, gap junctional coupling has not yet been detected in these cells (Xin and Bloomfield, 1997; Zhou, 1998).

Therefore, we herein investigated whether gap junctional coupling by SACs occurs during the developmental period in the mouse retina. The results obtained revealed gap junctional coupling between SACs and other cell types before eye-opening as well as decreases in coupling with postnatal development independent of visual experience.

## 2. Materials and methods

### 2.1. Animals

The IG-8 line of heterozygous transgenic mice (C57BL/6N), which express green fluorescent protein (GFP) signals in SACs in the retina under the control of the metabotropic glutamate receptor 2 promoter, was previously reported for this transgenic line (Watanabe et al., 1998; Yoshida et al., 2001). Transgenic mice were backcrossed to C57BL/6J mice. The mice of both sexes were used at postnatal day 3 (P3), P9, P15, and P28. Mice were housed with a 12:12-h light:dark cycle in a temperature-controlled room. Animals were euthanized by neck dislocation 2 h after lights on. Fresh water and a rodent diet were supplied *ad libitum*. When the dark rearing of pups was necessary, pregnant mice were reared in the dark before the birth of pups. Dark reared mice were euthanized with the same time schedule for mice housed with a 12:12-h light:dark cycle. The eyes were enucleated and hemisected, and the retinas were isolated from the sclera in Ringer’s solution (in mM: 115 NaCl, 5 KCl, 26 NaHCO_3_, 2 CaCl_2_, 1 MgCl_2_, 1.1 NaH_2_PO_4_, and 20 D-glucose, pH 7.4, bubbled with 95% O_2_ and 5% CO_2_). Isolated retinas were used for whole-mount, slice, or dissociated preparations.

### 2.2. Retinal whole-mount and slice preparations

Whole-mount retinas were incubated in Ringer’s solution at room temperature for > 30 min before patch-clamp recordings or dye injections. Details on the methods used for retinal slice preparation were previously described (Kaneda et al., 2008; Ishii and Kaneda, 2014; Ishii et al., 2017). In brief, isolated retinas were placed on a membrane filter (pore size, 0.45 μm; Advantec Toyo, Tokyo, Japan) with the retinal ganglion cell (RGC) side down and sliced at a thickness of 150 μm in Ringer’s solution. All experimental procedures were conducted at room temperature.

### 2.3. Patch-clamp recordings and the calculation of membrane capacitance (Cm)

Whole-cell recordings were made from ON- or OFF-type SACs in all retinal quadrants identified by a GFP fluorescent signal when viewed under a fluorescent microscope (BX51WI; Olympus, Tokyo, Japan). Retinas were superfused at a rate of 2 ml/min with Ringer’s solution. Data recordings were conducted in Ringer’s solution containing 1 μM SR95531 (Sigma-Aldrich, Taufkirchen, Germany) to block IPSCs at room temperature. Other drugs were also applied to the bath holding the retina. Meclofenamic acid (MFA) was purchased from Sigma-Aldrich and SKF38393 and SCH23390 from Nacalai Tesque, Inc (Kyoto, Japan). Patch pipettes with a resistance of 7–9 MΩ (when filled with an intracellular solution) were fabricated from borosilicate glass. The composition of the intracellular solution was as follows (in mM): 120 CsCl, 5 EGTA, 0.5 CaCl_2_, 10 HEPES, 5 ATP-2Na, and 1 GTP-3Na (pH adjusted to 7.2 with CsOH). Recordings were made with a patch clamp amplifier (Axopatch-200B; Molecular Devices, San Jose, CA, USA) connected to a Digidata 1322A interface and pCLAMP 10.3 software (Molecular Devices). Data were sampled at 10 kHz after passing a low-pass filter at 5 kHz. All recordings were started at least 5 min after achieving the whole-cell recording configuration.

**FIGURE 1:**
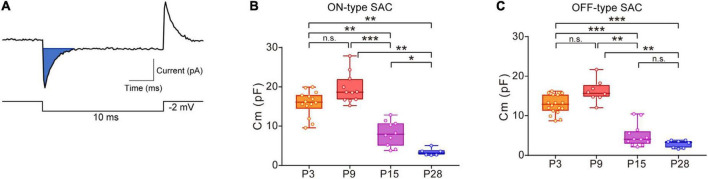
Developmental changes in membrane capacitance (Cm) in SACs. **(A)** Traces showing a capacitive current (top, blue area) to command the voltage step pulse (steps of –2 mV with a duration of 10 ms) (bottom). **(B,C)** Box plot of developmental changes in Cm in ON-type SACs **(B)** and OFF-type SACs **(C)**. Whiskers show maximum and minimum values, boxes show lower and upper quartiles, and horizontal lines correspond to the median. Each dot is an individual cell. The numbers of mice used were 4 (P3), 3 (P9), 3 (P15), and 4 (P28) for ON-type SACs, and 6 (P3), 3 (P9), 3 (P15), and 4 (P28) for OFF-type SACs. n.s., not significant; *p < 0.05; **p < 0.01; ***p < 0.001 (Kruskal–Wallis tests with the Steel-Dwass test).

The Cm of SACs was calculated according to a previous study (Lindau and Neher, 1988). The whole-cell current evoked by a step pulse (steps of −2 mV with a duration of 10 msec) at a holding potential of −70 mV was averaged (5–10 sweeps). Averaged capacitive currents were used to calculate Cm (Figure 1A, blue area). The effects of drugs (MFA, SKF38393, and SCH23390) on Cm were evaluated by comparing Cm recorded during and before (control) drug application. Cm for the control was calculated by averaging Cm during the last 5 min within the control, and Cm during drug application was calculated from the peak value for Cm during drug application.

### 2.4. Neurobiotin tracer coupling experiments

Whole-mount retinas were used for the dye injection. Since the cell bodies of OFF-type SACs were located in the inner nuclear layer in the whole-mount preparation, it was difficult to inject Neurobiotin into OFF-type SACs without damaging other cells. Therefore, experiments were only performed on ON-type SACs. Under the whole-cell clamp mode, ON-type SACs were held at −60 mV for 20 min. Electrodes were filled with Neurobiotin™ (Vector Laboratories, Burlingame, CA, USA, SP-1120) at ∼2% wt/vol and ∼290 mOsm in cesium chloride internal solution. Electrode resistance was 10∼12 MΩ. After tracer filling, the retina was fixed in 4% paraformaldehyde in 0.1 M phosphate buffer (PB) for 20 min. The retina was then incubated in 0.1 M PB containing 0.3% Triton-X 100 for 15 min. After the incubation in 0.1 M PB containing 1% Block Ace (Dainippon Pharmaceutical Co., Ltd., Osaka, Japan) for 1 h, samples were reacted with the primary antibodies, rabbit anti-RNA-binding protein with multiple splicing (RBPMS), a marker of RGCs (Kwong et al., 2010; Rodriguez et al., 2014) (working dilution, 1:500) (Abcam, Cambridge, MA, USA, ab194213), and rat anti-GFP (working dilution, 1:500) (Nacalai Tesque, 04404-84) in 0.1 M PB containing 0.4% Block Ace at 4°C for 48 h. Samples were then allowed to react with streptavidin-conjugated Alexa Fluor 594 (working dilution, 1:500) (Invitrogen, Carlsbad, CA, USA, S11227) and the secondary antibodies, Alexa Fluor 488-conjugated donkey anti-rat IgG (working dilution, 1:1,000) (Life Technologies, Carlsbad, CA, USA, A11006) and either Alexa Fluor 647-conjugated donkey anti-rabbit IgG (working dilution, 1:500) (Life Technologies, A31573) or Alexa Fluor Plus 405-conjugated goat anti-rabbit IgG (working dilution, 1:1,000) (Thermo Fisher Scientific, Waltham, MA, USA, A48254) in 0.1 M PB at room temperature for 2 h. Nuclear staining was performed with 4′,6-diamidino-2-phenylindole (DAPI) (Nacalai Tesque, 11034-56). The retina was mounted in Fluoro-KEEPER Antifade Reagent (Nacalai Tesque, 12593-64) for observations. Fluorescent images were captured using confocal microscopes (FV1200; Olympus, Tokyo, Japan, and LSM-980; Carl Zeiss, Jena, Germany).

### 2.5. Dissociation and sorting of retinal cells

Dissociated cells were prepared as previously described (Ishii et al., 2017). Briefly, the isolated retina obtained from P9 or P28 mice was incubated for 30–40 min in an external solution (which comprised, in mM, 135 NaCl, 5 KCl, 2 CaCl_2_, 1 MgCl_2_, 10 glucose, and 5 HEPES; pH adjusted to 7.4) containing 2.5 U/ml papain (Worthington Biochemical, Freehold, NJ, USA) and its activator, L-cysteine (0.1 mg/ml) bubbled with 100% O_2_ at 37°C. The enzyme treatment was stopped by washing the retina with the external solution containing 0.1 mg/ml of bovine serum albumin. The retina was then triturated with a Pasteur pipette. Retinal cells were re-suspended at a final concentration of 2–3 × 10^6^ cells/ml in the external solution containing 0.5 μg/ml propidium iodide. GFP-positive cells (1 × 10^4^ cells per sample) were collected by fluorescence-activated cell sorting (FACS) (Aria II, BD Biosciences, Franklin Lakes, NJ, USA) as SACs. Propidium iodide-positive cells were excluded as dead cells. The accuracy of the collection of GFP-positive cells was visually inspected by fluorescent microscopy. Scatter data were displayed by FlowJo_v10.7.2 (BD Biosciences). Collected GFP-positive cells were used in the gene expression analysis.

### 2.6. Gene expression analysis

Gene expression analyses were performed as previously described (Ishii et al., 2015), except for the methods of extracting RNA and reverse transcription. Total RNA was extracted using ISOSPIN Cell & Tissue RNA (Nippon Gene, Japan) from GFP-positive cells. Reverse transcription to obtain complementary DNA (cDNA) was conducted using ReverTra Ace qPCR RT Master Mix with gDNA Remover (Toyobo, Japan). The resulting cDNA was amplified with gene-specific primers and SYBR Premix Ex Taq II (TaKaRa, Japan). A real-time polymerase chain reaction analysis was performed using Thermal Cycler Dice Real-time System Single (TaKaRa). Reactions were performed at 95°C for 30 s, followed by 40 cycles at 95°C for 5 s and 60°C for 30 s. All processes were conducted according to the manufacturer’s instructions. Primer sequences, product sizes, and accession numbers are listed in Table 1.

**TABLE 1:** Primer sequences used for qPCR.

Gene		Primer sequence (5′–3′)	Size (bp)	Accession number
*Gje1* (Cx23)	Forward	cggccaataactccccaagt	136	NM_029722.2
	Reverse	tcacagtgtacacgggcttc		
*Gjd2* (Cx36)	Forward	cggtactgcccagtctttgt	174	NM_010290.2
	Reverse	gtctcccctacaatggccac		
*Gja1* (Cx43)	Forward	tggagatgcacctgaagcag	252	NM_010288.3
	Reverse	ttttctccgtgggacgtgag		
*Cjc1* (Cx45)	Forward	gagttctggtgaacagggca	297	NM_001159382.1
	Reverse	gggagttgcaaccaggatga		
*Gja8* (Cx50)	Forward	agacagcaccagtttctccg	133	NM_008123.3
	Reverse	aagagcactgtgagccagac		
*Drd1*	Forward	gtcttggtcatgccctggaa	135	NM_001291801.1
	Reverse	cacgctgatcacacagaggt		
*EGFP*	Forward	acgtaaacggccacaagttc	187	U57608
	Reverse	aagtcgtgctgcttcatgtg		

### 2.7. Statistical analysis

**FIGURE 2:**
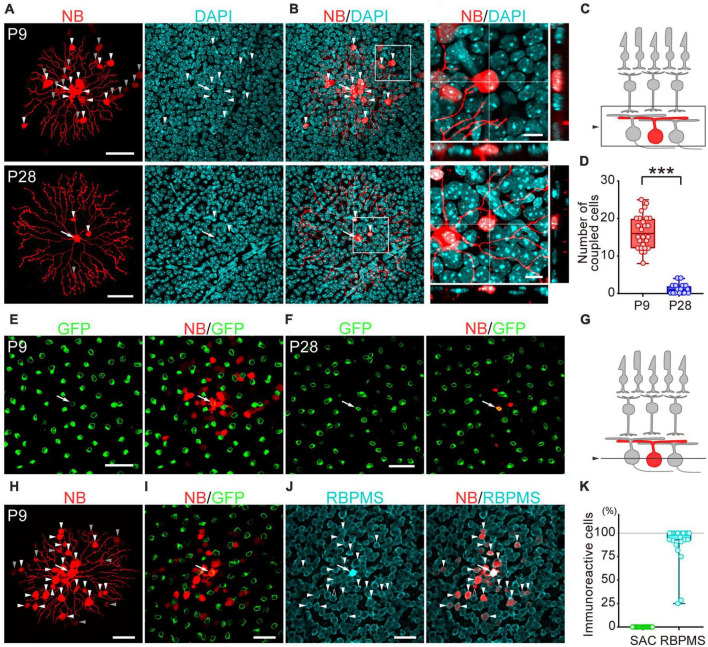
Developmental changes in the number of dye-coupled cells in ON-type SACs. Fluorescent images of Neurobiotin (NB)-injected ON-type SACs. **(A,H)** Projected images of NB-injected ON-type SACs (white arrow), brightly labeled dye-coupled cells (first-order direct connections, white arrowheads), and dimly labeled dye-coupled cells that may include second-order connections (gray arrowheads) at P9 and P28. Left: NB (red), middle: DAPI (cyan), right: merged image. **(B)** Higher magnification images within the white square of **(A)**. Cross-section images of horizontal and vertical line positions are also shown (bottom and right). **(C)** Schematic diagram of the projection range in **(A,B,H)**. The projected range is shown in the black rectangle. **(D)** Numbers of dye-coupled cells at P9 and P28. Each dot is the number of dye-coupled cells for each NB-injected SAC. ***p < 0.001 (the Mann–Whitney test). **(E,F,I,J)** Single optical sections of NB-injected ON-type SACs and dye-coupled cells in the ganglion cell layer (GCL). **(E,F)** Immunoreactivity for GFP (green) at P9 **(E)** and P28 **(F)**. NB-injected SACs (white arrow) and tracer-coupled cells are shown (red). These NB-injected cells are the same cells shown in the upper or lower panels of figure **(A)**, respectively. **(G)** Schematic diagram of the confocal plane in **(E,F,H,I)**. **(I,J)** Immunoreactivity for GFP (green) **(I)** or RBPMS (cyan) **(J)** at P9. NB-injected SACs (white arrow) and tracer-coupled cells are shown (red). The fluorescence of NB colocalized with the soma of RBPMS-immunoreactive cells is shown by arrowheads, while a cell not colocalized with RBPMS is shown by an empty arrowhead. NB-injected cells are the same cells shown in figure **(H)**. **(K)** Percentage of tracer-coupled cells with SAC or RBPMS at P9. Each dot is the percentage of dye-coupled cells with GFP or RBPMS immunoreactivity for each NB-injected SAC. The numbers of mice used were 13 (P9) and 9 (P28). Scale bars are 50 μm for **(A,E,F,H,I,J)**, and 10 μm for **(B)**.

**FIGURE 3:**
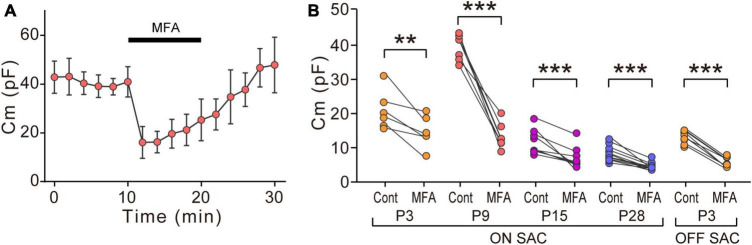
Effects of MFA on Cm in SACs. **(A)** Effects of 300 μM MFA on Cm in ON-type SACs at P9. Data represent the mean ± SD. **(B)** Summary of the effects of MFA on Cm at P3, P9, P15, and P28 in ON-type SACs and at P3 in OFF-type SACs. Control, the average of Cm values sampled 5–10 min after the start of experiments (before the application of MFA); MFA, the Cm value sampled when the effects of MFA peaked after its application. Each dot is an individual cell. The numbers of mice used were 5 (P3), 6 (P9), 4 (P15), and 6 (P28) for ON-type SACs, and 6 (P3) for OFF-type SACs. **p < 0.01; ***p < 0.001 (One-tailed paired *t*-test).

**FIGURE 4:**
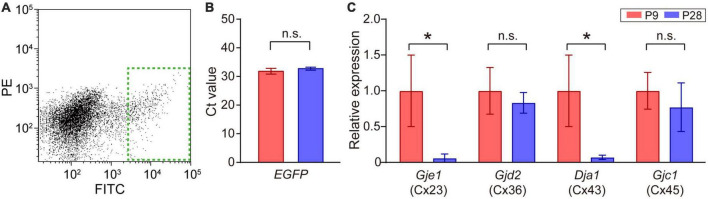
Quantitative analysis of connexin transcripts in SACs. **(A)** Collection of EGFP-labeled SACs from the mouse retina at P9 by FACS. The inside of the green dotted rectangle was sampled as EGFP-expressing SACs. FITC: fluorescein isothiocyanate, PE: phycoerythrin. **(B)** Threshold cycle (Ct) values of *EGFP* mRNA in SACs at P9 and P28. **(C)** Relative mRNA expression levels of connexin family members: *Gje1* (Cx23), *Gjd2* (Cx36), *Gja1* (Cx43), and *Gjc1* (Cx45). Data were normalized by the expression level of EGFP. The numbers of animals were 4 (P9) and 5 (P28). Data are shown as the mean ± SEM; n.s., not significant; *p < 0.05 (the Mann–Whitney test).

**FIGURE 5:**
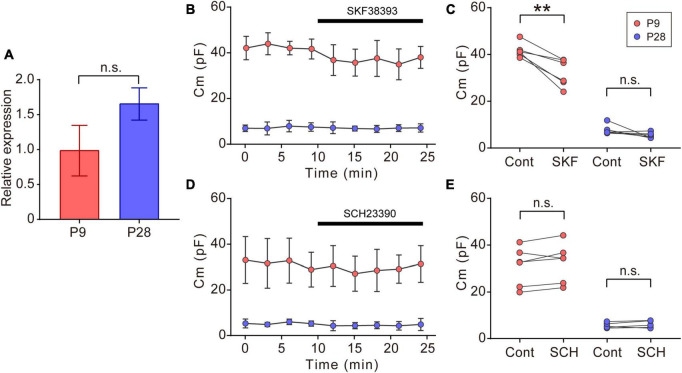
Effects of dopaminergic inputs on Cm in ON-type SACs. **(A)** Relative mRNA expression levels of *Drd1* in SACs at P9 and P28. The mRNA expression level of *EGFP* was used as an internal standard. EGFP-expressing cells were collected using flow cytometry. The numbers of animals were 4 (P9) and 5 (P28). Data are shown as the mean ± SEM. n.s., not significant (the Mann–Whitney test). **(B,D)** Effects of 10 μM SKF38393 (SKF, **B**) and 10 μM SCH23390 (SCH, **D**) on Cm at P9 (red circle) and P28 (blue circle). Data are shown as the mean ± SD. **(C,E)** Summary of the effects of SKF38393 and SCH23390 on Cm. Control, average Cm values sampled 5–10 min after the start of experiments (before drug application); SKF and SCH, the Cm value when the effects of SKF or SCH peaked during drug application. Each dot is an individual cell. The numbers of mice used were 4 (P9) and 4 (P28) for the SKF application, and 3 (P9) and 3 (P28) for the SCH application. Data in **(B,D)** represent the mean ± SD; n.s., not significant; **p < 0.01 (One-tailed paired *t*-test).

**FIGURE 6:**
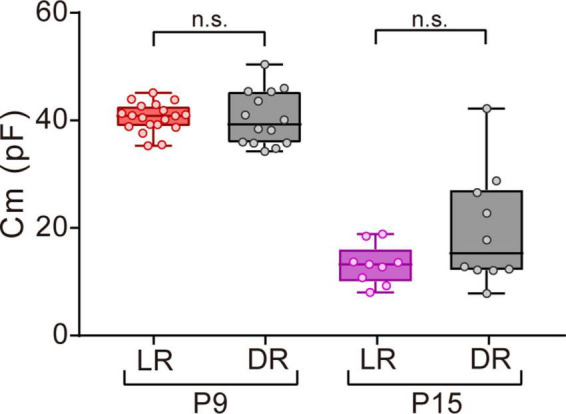
Effects of dark rearing on Cm in ON-type SACs. Effects of dark rearing on Cm in ON-type SACs at P9 and P15. The Cm for each condition is shown by a box plot. Whiskers show maximum and minimum values, boxes show lower and upper quartiles, and horizontal lines correspond to the median. Each dot is an individual cell. The numbers of mice used were 6 (LR) and 3 (DR) at P9 and 5 (LR) and 3 (DR) at P15. LR, mice reared under normal light/dark cycle conditions; DR, mice reared under dark conditions; n.s., not significant (the Mann–Whitney test).

Statistical analyses were performed using Prism 7.0 (GraphPad Software, La Jolla, CA, USA) and R (version 4.1.1) (Ihaka and Gentleman, 1996). Grouped data in Figures 1, 2, 6 were presented in box plots, with the central line showing the median and the lower and upper edges of the box indicating 25 and 75% of data, respectively. We used a parametric or non-parametric test depending on the results of normality and homoscedasticity tests. If any dataset did not exhibit a normal distribution or homoscedasticity, non-parametric tests were performed. Kruskal–Wallis tests were used for multiple comparisons with the Steel-Dwass test to examine the significance of differences (see Figure 1). The Mann–Whitney test was used when two unpaired groups were compared (see Figures 2, 4, 5, 6). Comparisons between recordings from the same cell before and after the application of pharmacological agents were performed using paired *t*-tests (see Figures 3, 5). Details on the results of individual statistical analyses are described in the figure legends.

## 3. Results

### 3.1. Developmental changes in Cm in ON- and OFF-type SACs

In ON- and OFF-type SACs, a change was observed in Cm during development. In ON-type SACs, the average Cm recorded in the slice preparation was 16.6 pF at P3 (Figure 1B). Average Cm peaked at P9 and then decreased to 3.4 pF by P28. Changes in Cm in OFF-type SACs were similar to those in ON-type SACs (Figure 1C). In biological membranes, the Cm of the unit membrane area was 1 μF/cm^2^ and was independent of the cell type (Hille, 1992). Under this condition, when we assume that SAC is a spherical cell with a soma diameter of 10 μm (Taylor and Smith, 2012), the estimated Cm of single SAC is ∼3 pF. The decrease observed in Cm after eye-opening was in contrast to previous findings showing that the soma size of SACs did not significantly differ during postnatal development (at P5, P10, P15, P20, P25, and P30) (Zhang et al., 2005) and also that an elongation of the dendrite length of SACs, indicating an increase in biological membranes, occurred throughout development (Wong and Collin, 1989). Therefore, large Cm at P3 and P9 implied that SACs were electrically coupled with other cells via gap junctions in the early postnatal stage.

### 3.2. Anatomical identification of gap junctional coupling

To examine whether gap junctional coupling formed between SACs and other cells in the early postnatal stage, the Neurobiotin tracer was injected into ON-type SACs (white arrow, Figures 2A, E, F, H–J) to visualize cells that coupled with ON-type SACs. Many dye-coupled cells were detected at P9 (Figures 2A, H). Some dye-coupled cells showed strong fluorescence (white arrowheads), while others showed weak fluorescence (gray arrowheads). In the present study, dye-coupled cells with weak fluorescence were excluded from analyses because they may have included second-order connections. Dye-coupled cells were radially distributed around dye-injected SACs (16.5 ± 4.5 cells, mean ± SD). On the other hand, only a few dye-coupled cells with small soma were observed at P28 (1.2 ± 1.4 cells, mean ± SD) (Figures 2A, B). The colocalization of DAPI, a nuclear marker, was observed in dye-coupled cells, indicating that Neurobiotin signals did not reflect the varicosities of SAC dendrites. The number of dye-coupled cells was significantly lower at P28 than at P9 (Figure 2D). At P9, dye-coupled cells did not overlap with GFP, a marker of SACs (Figures 2E, I, K), whereas the majority of dye-coupled cells showed immunoreactivity for RBPMS, a marker of RGCs (Figures 2J, K, white arrowheads). At P28, dye-coupled cells did not overlap with GFP. However, further identification was not performed because of the limited number of dye-coupled cells. These results suggest that large Cm in SACs was due to the formation of electrical coupling via gap junctions and that dye-coupled cells in ON-type SACs were heterologous, not homologous cells.

### 3.3. Effects of MFA on Cm in ON- and OFF-type SACs

We examined the effects of MFA, a gap junction inhibitor, on Cm. In ON-type SACs, the application of 300 μM MFA to a whole-mount preparation significantly reduced Cm at P9 (Figures 3A, B). The inhibitory effects of MFA on Cm peaked within 2 min and persisted during the application of MFA. After the washout of MFA, Cm recovered to the control level. The inhibitory effects of MFA on Cm were also observed at P3, P15, and P28 (Figure 3B). In OFF-type SACs, we investigated the effects of MFA on Cm at P3 only because OFF-type SACs in the slice preparation at P9 deteriorated and did not recover after the application of MFA. In OFF-type SACs at P3, we observed a significant decrease in Cm after the application of MFA (Figure 3B).

### 3.4. Gene expression of connexin families in SACs

The retina uses multiple types of connexins to form various types of gap junctions (Guldenagel et al., 2000; Volgyi et al., 2013). The expression of multiple types of connexin genes has also been reported in SACs (Yan et al., 2020). Therefore, we investigated whether a postnatal change occurred in the expression levels of connexin genes [connexin 23 (Cx23), 36 (Cx36), 43 (Cx43), 45 (Cx45), and 50 (Cx50)] (Table 1) in SACs. A fraction of SACs was separated as GFP-positive cells by FACS (Figure 4A, dotted green rectangle). The Ct value of *EGFP* mRNA, the internal standard in the present study, did not significantly differ between P9 and P28 (Figure 4B). All connexins examined were detected at P9. However, Cx50 was detected in 2 out of 8 samples. The relative expression of Cx23 and Cx43 was significantly lower at P28 than at P9, whereas that of Cx36 and Cx45 did not significantly differ between P9 and P28 (Figure 4C).

### 3.5. Effects of dopamine on Cm in ON-type SACs

In the vertebrate retina, dopamine is synthesized and released from dopaminergic amacrine cells (Roy and Field, 2019). Dopamine release is detected before eye-opening and increases after eye-opening (Melamed et al., 1986; Casini et al., 2004). The dopamine D1 receptor (D1R) regulates gap junctional coupling between retinal neurons (Bloomfield and Volgyi, 2009). Moreover, SACs have been reported to express *Drd1* encoding D1R at P19 (Yan et al., 2020). In our experiments, GFP-positive cells also expressed *Drd1* at P9 and P28 (Figure 5A). Therefore, we investigated whether dopamine regulated gap junctional coupling in ON-type SACs using a whole-mount preparation. The application of 10 μM SKF38393, a D1R agonist, slightly reduced Cm at P9, but did not exert any effects on Cm at P28 (Figures 5B, C). On the other hand, the application of 10 μM SCH23390, a D1R antagonist, did not increase Cm at P9 or P28 (Figures 5D, E). The present results suggest that gap junctional coupling by SACs in the early postnatal stage was regulated by D1R, similar to other gap junctional coupling types in the retina (Hampson et al., 1992; Yadav et al., 2019; Banerjee et al., 2020).

### 3.6. Effects of visual experience on Cm in ON-type SACs

Visual experience has been reported to play an important role in functional and morphological maturation in the retina (Sernagor and Grzywacz, 1996; Zhang et al., 2005; Tian, 2008). Since mice open their eyes by P12–14 (Ford and Feller, 2012; Hoy and Niell, 2015), we investigated whether the deprivation of visual experience retarded the maturation process of gap junctional coupling by SACs. We compared the Cm of ON-type SACs between normal light/dark cycle-reared and dark-reared mice at P9 (before eye-opening) and P15 (after eye-opening) using a whole-mount preparation. No significant differences were observed in Cm between normal light/dark cycle- and dark-reared mice at both ages (Figure 6), whereas variance at P15 was larger in dark-reared mice (112.8) than in normal light/dark cycle-reared mice (13.7) (*p* < 0.01, the F-test). This result suggests that the deprivation of visual experience did not retard the maturation process of gap junctional coupling by SACs.

## 4. Discussion

We herein demonstrated the formation of gap junctional coupling between SACs and heterologous cells (the majority of cells were RGCs), but not homologous cells (SACs) in the early postnatal stage. Gap junctional coupling started to disappear between P9 and P15 and mostly disappeared by P28. Gene expression levels among connexin family members in SACs also changed between P9 and P28. The remodeling process of gap junctional coupling in the early postnatal stage was not affected by visual experience. In the early developmental stage, gap junctions play an important role in synaptogenesis as well as in the formation of neural circuits and the maturation of the retina (Cook and Becker, 2009; Arroyo et al., 2016). Therefore, gap junctional coupling by SACs in the early developmental stage may be necessary for the formation of neural circuits in the retina.

Gap junctional coupling by SACs started to decrease between P9 and P15 and mostly disappeared by P28 in ON-type SACs. Gap junctional coupling between ON-type SAC and RBPMS-positive cells was observed in the early developmental period. In the retina, RBPMS is selectively expressed in RGCs (Kwong et al., 2010; Rodriguez et al., 2014), including ON-OFF type direction-selective RGCs (DSGCs) (Dhande et al., 2019). ON-OFF DSGCs have been shown to form gap junctions with homologous (ON-OFF DSGCs) or heterologous cells (other cell types) in the mouse retina (Xu et al., 2013). Gap junctional coupling in ON-OFF DSGCs also started to disappear at a similar developmental stage (between P12 and P15), suggesting the potential of SACs as candidate heterologous cells for ON-OFF DSGCs. In the cortex, gap junctional coupling in the early stage of development is necessary for the formation of chemical synapses (Pereda, 2014). Since ON-type SACs form cholinergic and GABAergic synapses with ON-OFF type DSGCs (Lee et al., 2010), gap junctional coupling in the early stage of development in SAC may function as a signal to guide the formation of chemical synapses between SACs and DSGCs.

At the mRNA level, we detected 5 subtypes (Cx23, Cx36, Cx43, Cx45, and Cx50) of the connexin family at P9 and observed reductions in Cx23 and Cx43 expression levels between P9 and P28 in GFP-positive cells (SACs). A previous study reported that Cx23 did not form gap junctions in mice (Sonntag et al., 2009). Therefore, Cx43 appears to function in the formation of gap junctional coupling by SACs in the early postnatal stage. This speculation is supported by findings showing that the activity of gap junctional coupling in ON-type SACs was controlled by D1R, a regulator of Cx36 and Cx43 (Urschel et al., 2006; Bosson et al., 2015). In addition, the application of MFA, a blocker of Cx36, Cx43, and Cx50 (Manjarrez-Marmolejo and Franco-Perez, 2016), reduced Cm by ∼9 pF at P9. However, reduced Cm was still larger than expected at the single cell level (∼3 pF), suggesting that connexin subtypes other than Cx43 also contributed to the formation of gap junctional coupling at P9.

In the present study, we detected connexin family members at the mRNA level using isolated SACs. According to the dataset of a single-cell transcriptomic analysis (Yan et al., 2020), the mRNAs of Cx36 and Cx45 were detected in some choline acetyltransferase (ChAT)-expressing cells at P19 (∼20% for Cx36 and ∼10% for Cx45), supporting the presence of Cx36 and Cx45 at P28. On the other hand, a developmental study on connexin subtypes (Cx36, Cx43, and Cx45) in the mouse retina reported different findings from the present results (Kihara et al., 2006; Kovacs-Oller et al., 2014). Since the previous study used the whole retina, which contains various retinal neurons and glial cells, the discrepancies between their findings and the present results reflect differences in the developmental patterns of connexin subtypes among retinal cells. At the protein level, previous studies reported the expression of Cx36 and Cx45 in the inner plexiform layer, including other retinal layers (Dedek et al., 2006; Kihara et al., 2006; Kovacs-Oller et al., 2014); however, the colocalization of a reporter protein with ChAT was not detected at P8 in Cx36 knock-in mice (Hansen et al., 2005). Furthermore, although the broad expression of Cx43 has been reported in the inner plexiform layer (Ivanova et al., 2019), it has also been detected in glial cells and vascular pericytes in the adult retina (Moore and O’Brien, 2015; Ivanova et al., 2019; Szarka et al., 2021; Toychiev et al., 2021). Therefore, further studies are warranted on the expression of C36, Cx43, and Cx45 throughout the developmental period in SACs.

Our Neurobiotin tracer study on SACs revealed gap junctional coupling in an early postnatal stage. However, when Lucifer Yellow, Alexa 488 Hydrazide, or Alexa 555 Hydrazide was used to visualize the morphology of SACs in neonatal animals (Wong and Collin, 1989; Sandmann et al., 1997; Zhou, 1998; Wei et al., 2011; Xu et al., 2016), gap junctional coupling was not detected. This difference may be attributed to the molecular sizes of tracers. The molecular weight (MW) of Neurobiotin (MW 323) is smaller than those of Lucifer Yellow (MW 457), Alexa 488 Hydrazide (MW 570), and Alexa 555 Hydrazide (MW 1150). We did not detect cells coupled with SACs using Alexa 594 Hydrazide (MW 759). Regarding homomeric connexin channels, the order of permeability is Cx36 < Cx45 < Cx43 (Saez et al., 2005). Among these connexins, Cx43 and Cx45 formed heteromeric channels with a similar permeability to homomeric Cx45 channels (Martinez et al., 2002), while Cx36 did not form heteromeric channels (Bedner et al., 2012). Since homomeric Cx43 channels are permeable to Neurobiotin and Lucifer Yellow and heteromeric Cx43-Cx45 channels to Neurobiotin alone (Martinez et al., 2002), SACs may have heteromeric Cx43-Cx45 channels before eye-opening.

At P9 and P28, we detected the expression of Cx36 and Cx45 and noted a decrease in Cm by MFA, a blocker of Cx36, Cx43, and Cx50 (Manjarrez-Marmolejo and Franco-Perez, 2016). Therefore, Cx36 is a likely candidate. However, gap junctional coupling at P28 was not controlled by dopaminergic inputs even though the dopaminergic regulation of Cx36 has been reported in the adult mouse retina (Urschel et al., 2006). This result showed that D1R was not functional by P28. In the adult mouse retina, D1R predominantly localizes to bipolar cells (Farshi et al., 2016), suggesting that SACs do not express D1R; however, we detected D1R at the mRNA level at P28 (Yan et al., 2020). On the other hand, we observed D1R at the mRNA level at P9 and also found that SKF38393 decreased the Cm of ON-type SAC, whereas SCH23390 had no effect on Cm. These results suggest that D1R modulated Cx36 at P9.

We used Cm as an indicator of the gap junctional coupling strength (de Roos et al., 1996) and observed changes in Cm during development. According to the cable theory, Cm is expressed by the equation τ = RmCm, where τ is membrane time constant, and Rm is the membrane resistance (Junge, 1992). Therefore, Cm is not determined by gap junctional coupling strength alone. Indeed, Cm is affected by the other factors such as various ion channels (Courjaret et al., 2016; Bigiani et al., 2022) and transporters (Gustafsson et al., 2015). Since neurons alter the expression levels of the other factors during development (Bando et al., 2022), we have to consider the possible contribution of the other factors to the changes in Cm. However, we speculate that the contribution of the other factors is not likely in the present study since the presence of gap junctional couplings has been demonstrated by tracer coupling experiments and the expression of connexin mRNA in SACs. A similar discussion may also be applicable to rule out the possibility that reduction of Cm by MFA is mediated by the off-target effects of MFA since previous studies demonstrated that MFA also functioned as an opener of KCNQ (Kv7) (Peretz et al., 2005) and ATP-sensitive K channels (Li et al., 2007). The absence of off-target effects by MFA is further supported by the previous reports that SACs mainly express Kv3 (Ozaita et al., 2004; Kaneda et al., 2007) and TREK1 (Ford et al., 2013) and the fact that there is currently no evidence to support the presence of KCNQ and ATP-sensitive K channels in SACs.

The present results showed that dark rearing did not change the time course of the disappearance of gap junctional coupling in SACs. In SACs, dark rearing did not affect the expression patterns of P2X2-purinoceptors (Kaneda et al., 2010) or P2Y1-purinoceptors (Dilip et al., 2013) or the size of soma (Zhang et al., 2005). In ON-OFF DSGCs, visual deprivation did not change the morphology of dendrites, tracer coupling patterns, receptive field properties, or direction selectivity (Chan and Chiao, 2008; Chen et al., 2009; Tiriac et al., 2022). These findings suggest that visual experience does not affect circuit formation by SACs in the developing retina. On the other hand, dark rearing until adulthood reduced the number of ChAT-immunoreactive cells (Zhang et al., 2005) and GABA-immunoreactive cells (Lee et al., 2006). The reduction in GABA immunoreactivity may be recovered at the protein level by the cessation of dark rearing in adulthood (Lee et al., 2006). Therefore, visual experience may modify the amount of transmitter released by SACs without affecting direction selectivity. Similarly, dark rearing may affect gap junctional coupling at the function level. In a previous study, connexin expression levels showed circadian rhythms; they were high at night and low in the day (Katti et al., 2013). In addition, connexin expression levels markedly varied at night. The larger variance of Cm in dark-reared mice than in light/dark cycle-reared mice at P15 suggests that visual experience reflects the loss of the proper control of connexin gene expression in the day and at night.

We used FACS-collected SACs, which are a mixture of ON- and OFF-type SACs, and detected Cx23, Cx36, Cx43, and Cx45 during postnatal development. We previously reported differences in the distribution of receptors between ON- and OFF-type SACs (Kaneda et al., 2004, 2008; Ishii and Kaneda, 2014; Ishii et al., 2017). Our findings were confirmed in recent studies (Whitney et al., 2014; Jain et al., 2022). According to a single-cell transcriptomic analysis, the expression of connexin subtypes varies from cell to cell (Yan et al., 2020) and the retina also expresses other subtypes of connexins (Guldenagel et al., 2000; Volgyi et al., 2013). Therefore, further studies are warranted to establish whether ON- and OFF-type SACs use different connexin subtypes for gap junctional coupling.

## Data availability statement

The raw data supporting the conclusions of this article will be made available by the authors, without undue reservation.

## Ethics statement

All experimental procedures were approved by the Animal Experiments Ethical Review Committee of Nippon Medical School (#2020-012).

## Author contributions

TM, TI, and MK designed the project, wrote the manuscript, and performed the patch-clamp analysis. TM and TI conducted immunohistochemistry. TI performed the gene expression analysis. All authors read and approved the final manuscript.
